# Costs and Benefits to Pregnant Male Pipefish Caring for Broods of Different Sizes

**DOI:** 10.1371/journal.pone.0156484

**Published:** 2016-05-31

**Authors:** Gry Sagebakken, Ingrid Ahnesjö, Charlotta Kvarnemo

**Affiliations:** 1 Department of Biological and Environmental Sciences, University of Gothenburg, Gothenburg, Sweden; 2 Department of Ecology and Genetics, Uppsala University, Uppsala, Sweden; University of Basel, SWITZERLAND

## Abstract

Trade-offs between brood size and offspring size, offspring survival, parental condition or parental survival are classic assumptions in life history biology. A reduction in brood size may lessen these costs of care, but offspring mortality can also result in an energetic gain, if parents are able to utilize the nutrients from the demised young. Males of the broad-nosed pipefish (*Syngnathus typhle*) care for the offspring by brooding embryos in a brood pouch. Brooding males can absorb nutrients that emanate from embryos, and there is often a reduction in offspring number over the brooding period. In this study, using two experimentally determined brood sizes (partially and fully filled brood pouches), we found that full broods resulted in larger number of developing offspring, despite significantly higher absolute and relative embryo mortality, compared to partial broods. Male survival was also affected by brood size, with males caring for full broods having poorer survival, an effect that together with the reduced embryo survival was found to negate the benefit of large broods. We found that embryo mortality was lower when the brooding males were in good initial condition, that embryos in broods with low embryo mortality weighed more, and surprisingly, that males in higher initial condition had embryos of lower weight. Brood size, however, did not affect embryo weight. Male final condition, but not initial condition, correlated with higher male survival. Taken together, our results show costs and benefits of caring for large brood sizes, where the numerical benefits come with costs in terms of both embryo survival and survival of the brooding father, effects that are often mediated via male condition.

## Introduction

Caring for a large number of offspring at the same time can obviously be beneficial for fitness, if done successfully. However, as costs of parental care often increase with offspring number, brood size is expected to be traded-off against the parent’s current and/or future reproductive success [1–3]. Hence, benefits of caring for a smaller brood include higher offspring weight and higher offspring survival, due to limited resources available to the brood [4, 5]. Another benefit of caring for a smaller brood can be an increased chance of the caring parent(s) to breed successfully again [5, 6]. However, the optimal brood size may depend on the caring parent’s age, size and condition [7, 8], offspring competition [9], as well as the surrounding environment of the brood, such as food availability, temperature and oxygen richness [10–12].

Protection of a brood against predators is a form of care that can be shared by many offspring without diminishing in value. It is therefore called shareable care [13]. In contrast, providing unshareable care (nutrients, oxygen, etc) entails increasing costs with increasing brood size for both parents and offspring [13–15]. When the resources provided by the parent(s) are limited, a reduced brood size can be a good option, as it will reduce sibling competition [16] by increasing the resources available towards each offspring [9]. Hence, offspring from a brood of reduced size might have better future fitness prospects than offspring from a full brood if resources are limited. However, individual offspring from a brood of reduced size may not necessarily receive more care than individual offspring of a full brood, as the parent may benefit from saving resources for future reproduction, by retaining resources to increase its condition and/or chance of survival [17]. A classic example of parents (re)gaining resources for current or future reproduction by brood size reduction is when a parent eats some or all of the young in a brood, a phenomenon called filial cannibalism [9,18–20]. Hence, parents in poor condition are expected to provide less care and/or to eat more of their offspring [18, 21].

Parental care may cause parental condition to deteriorate to such an extent that the parent dies [22]. In the river bullhead (*Cottus gobio*) there is nearly a tenfold increase in death rate in males (the caring sex) when they are taking care of eggs, linked to a deterioration of body condition [23]. Such deterioration of body condition, reducing the caring parent’s survival, might be caused by higher energy expenditures or physiological stress and is reported in several fish species [22]. In the broad-nosed pipefish (*Syngnathus typhle*), for example, survival among males and females kept from mating was significantly higher than among individuals allowed to reproduce i.e. egg-spawning females and brooding males [24].

**Fig 1:**
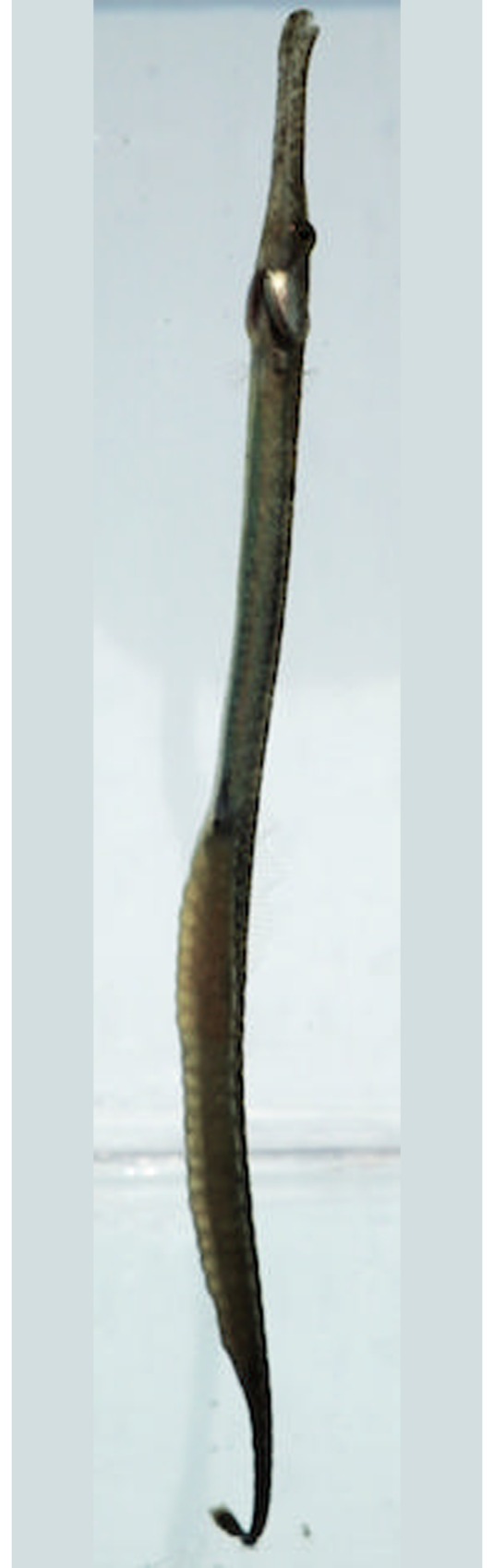
A pregnant pipefish male. A male of the broad-nosed pipefish (*Sygnathus typhle*) with a full brood pouch. Note the thin pouch folds that allow the eggs to be counted though the brood pouch in newly mated males.

In this study, we aimed to examine the potential benefits of a reduced brood size on the individuals in the brood and the caring father in the broad-nosed pipefish (*S*. *typhle*) (Fig 1). Like other seahorses and pipefishes, this species shows exclusive male care [25]. At mating, the female transfers eggs into the male’s brood pouch, the eggs are fertilized and the resulting embryos are not only protected and osmoregulated inside the pouch, but the brood also receives nourishment and oxygenation [25–29]. The brooding period lasts for five to eight weeks, depending on temperature [30], and the male broods the embryos until they are released as independent young [25, 31]. The pipefish life span is 2 or even 3 years, although most individuals only survive one breeding season, and both sexes breed multiple times during each season [24, 32, 33], prompting trade-offs between current and future reproduction [34]. Furthermore, in the study population, the reproduction is characterized by female mating competition for male partners, as females on average produce more eggs than males are able to brood during the same period [30, 32–33].

A reduction in embryo number during brooding is common in the focal pipefish population, as is well documented both in the field and the laboratory [35–38], and it has been found in several other pipefish species as well [34, 39]. At present, the factors affecting embryo mortality in the pipefish brood pouch are only partially understood. Using radioactive labelling, we have shown that nutrients that originate from embryos can be absorbed by the brooding father and incorporated into his body tissues, such as liver and muscle tissue [40]. Thus, the father not only provides nutrients to the developing young [26], but is also able to take up resources of maternal origin during brooding, in a way that conceptually resembles filial cannibalism. We also know that larger embryos have higher chance of survival until birth [36] and that embryos in half-sib broods have higher survival compared to full-sib broods [38]. Furthermore, embryo survival correlates positively and significantly with male condition [41].

In this study, we determined the brood size experimentally and examined the effects of reduced brood size on different life history fitness parameters, such as embryo survival, embryo weight, paternal survival and paternal condition, with full brood size as control. We thus allowed one female to either fill up the male’s brood pouch with eggs completely or to fill approximately half of the brood pouch with eggs, resulting in two treatments and the following predictions: (1) If brood size is linked to embryo mortality within a brood, we expect partially filled males to show lower levels of embryo mortality than fully filled males. (2) We also expect higher embryo mortality in brooding males of poorer initial condition, if paternal care constitutes an energy-limited resource. (3) We expect embryo weight to be higher in a partial brood compared to a full brood as parental care resources are limited and these resources have to be divided between more offspring in the full brood compared to the partial brood. Furthermore, we expect (4) lower male mortality and higher male final condition in males caring for a partial brood compared to males caring for a full brood. However, (5) if costs of care are unrelated to brood size, we expect no difference in relative embryo mortality, embryo weight, male mortality and male condition between the two treatments. Our laboratory-based study was carried out in a semi-natural setting, with natural seawater, live prey and naturally occurring pathogens.

## Materials and Methods

The study was carried out at the Sven Lovén Centre for Marine Sciences, Kristineberg, at the west coast of Sweden (58°15'N, 11°28'E), from April to June 2009. Our work complies with the international animal care guidelines of the Association for the Study of Animal Behaviour [42] and the ARRIVE guidelines [43]. The study also meets the national legal requirements of Sweden: it was approved by the Ethical Committee for Animal Research in Gothenburg (permit number dnr 111–2007) and carried out in facilities approved by the Ministry of Agriculture (dnr 31-4061/2008). No other permits were required for the location and activity, and the study did not involve endangered or protected species.

The broad-nosed pipefish (*Syngnathus typhle*) were caught from end of April to early May, before the onset of their breeding season. The fish were captured in meadows of eelgrass, *Zostera marina*, nearby the station in <10 m deep bays of the Gullmar Fjord, using a small beam trawl, 4 mm mesh size, pulled by a boat, or by a handheld beach seine in shallow water. The fish were separated by sex and size in the laboratory and kept in 200 l barrels containing artificial *Zostera*, air stones, continuously renewed natural seawater at 15°C, and with artificial light on from 06:00 to 23:00. Cultivated brine shrimp (*Artemia sp*.) and wild caught crustaceans (*Crangon crangon*, *Praunus flexuosus* and *Copepoda sp*.*)* were fed to the fish *ad libitum* three times a day.

Each male was randomly assigned to one of two treatments. Before he was placed in a 70 l mating aquarium with one female, standard length (SL) was measured on a measuring board to the nearest mm and body width was measured across the abdomen using calipers to the nearest 0.1 mm. In the fully filled treatment (FF) each male was allowed to mate with one female until his brood pouch was completely filled with eggs, whereas in the partially filled treatment (PF), mating was interrupted, with a dip net in the tank, when the male’s brood pouch was estimated to be 50 percent filled. In all trials the female was removed from the aquarium with a dip net, the brood size after mating was left unaltered, and no eggs were removed from the pouch. Each female (N = 74) was mated to one male and then released back into the bay where it had been caught. Medium sized females, range 176–205 mm SL, were used to limit egg size variation in the experiment, as egg size and female size are positively correlated in this population [44–45].

**Fig 2:**
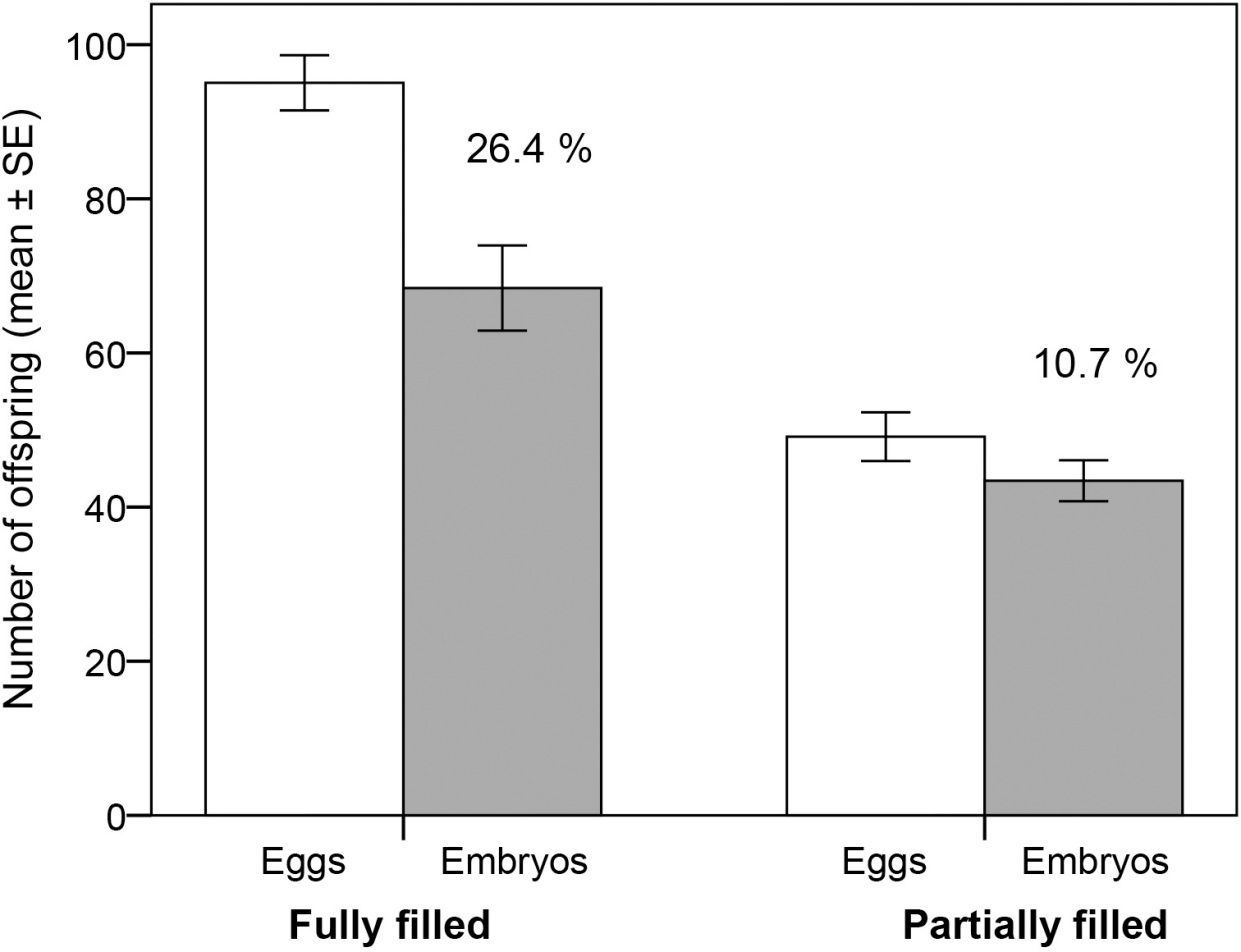
Brood size and embryo mortality. Mean number (± SE) of eggs at the time of mating (open bars) and of embryos (shaded bars) after approx. 50% of the brooding period in broad-nosed pipefish males (*Syngnathus typhle*) with brood pouches filled partially (N = 36) or fully (N = 38). Percent embryo mortality is shown for each of the two treatments.

The eggs were counted after mating, through the males’ thin pouch walls (Fig 1). On average, PF males received 49.1 ± 3.2 eggs and FF males 95.1 ± 3.6 eggs (Fig 2), which is significantly different (independent t-test: t = 9.58, N = 74, P < 0.001). To count the eggs, each male was sedated in water with 2-phenoxyethanol (100 μl l^-1^) and the number of eggs was counted, while using a cold light shone through the pouch. While sedated, the males were also individually color marked with non-toxic latex paint (Liquitex^®^, USA) on their belly and with a carbon-based black tattoo dot on their tail to provide individual identification. The fish were then allowed to recover in a bucket of fresh seawater. The whole handling, from sedation to recovery took less than 5 minutes. Both marking methods are well tested [37, 46–47]. The tattoo needles consisted of 5 short fine metal fibers mounted in a tight bundle, which allowed the ink to enter the skin to the intended depth, but not deeper. The latex paint was diluted with a small amount of water to make it more fluid before it was injected subcutaneously using a fine syringe. After marking, the PF and FF males were placed randomly into one of four different 400 l basins with a density of approximately 18 l fish ^-1^. There was no difference in fish numbers between the basins (χ^2^ = 1.17, df = 3, P = 0.76). All basins had the same feeding regime and environmental conditions as mentioned above, and mortality did not differ between the basins (Fishers exact: P = 0.41).

There were 36 males in the PF group and 38 males in the FF group. On average, the PF males were 156.4 ± 2.3 mm SL and 4.3 ± 0.08 mm in trunk width. FF males were on average 159.3 ± 2.2 mm SL and 4.3 ± 0.08 mm in trunk width. The two groups of males did not differ in length (one-way ANOVA: F_1,72_ = 0.80, P = 0.37) or trunk width (one-way ANOVA: F_1,72_ = 0.001, P = 0.98). An initial condition index was calculated as the residuals of male trunk width regressed on male SL. We found no difference between the two groups in initial condition index (one-way ANOVA: F_1,72_ = 0.61, P = 0.44). Females mated to PF males were, on average, 188.9 ± 1.2 mm SL and had a trunk width of 7.8 ± 0.17 mm before mating. Females mated to FF males were, on average, 189.2 ± 1.1 mm SL and had a trunk width of 7.8 ± 0.12 mm. The two groups of females did not differ in length (one-way ANOVA: F_1,72_ = 0.03, P = 0.87) or trunk width (one-way ANOVA: F_1,72_ = 0.02, P = 0.89). We also calculated residuals of female trunk width regressed on female SL, and found no difference between the two groups (one-way ANOVA: F_1,72_ = 0.06, P = 0.81).

On average, males in the PF group brooded for 19.5 ± 1.2 days and males in the FF group for 19.0 ± 1.2 days, which correspond to 55 percent of a full gestation time at 15°C [30]. Based on daily inspections, 38 of 74 (51%) of the brooding males showed signs of terminal illness, most often due to fungal or skin infections (also common in the field during the same time period), or the fish being unable to regulate the swim bladder. These signs were used as criteria for euthanasia, following the protocol in our ethical permit, using an overdose of 2-phenoxyethanol (2 ml l^-1^). Hence, as these fish died before the end of the trial, this subset of males brooded for on average 14.1 ± 0.9 days (range 3–28 days), whereas the rest (N = 36) brooded for 24.7 ± 0.6 days (range 12–32 days; this range of brooding times was created to match the fact that the males that died prematurely brooded for varying numbers of days). Brooding time did not differ between PF and FF males in the whole data set, or among the surviving males (all males: one-way ANOVA: F_1,72_ = 0.12, P = 0.73; survivors only: one-way ANOVA: F_1,34_ = 0.56, P = 0.46). At the end of the experiment the remaining males were also killed using an overdose of 2-phenoxyethanol (2 ml l^-1^). All males were preserved in 70 percent ethanol for later dissections.

At dissection, all embryos were taken out of the brood pouch and counted. Embryo numbers were then compared to the initial number of eggs that the male received at mating, providing a measurement of absolute (number of eggs at mating–number of embryos) and relative embryo mortality [(number of eggs at mating–number of embryos) x egg number at mating^-1^]. To determine embryo weight, we took 10 embryos from the lower half of the pouch and 10 embryos from the upper half of the pouch (when the pouch had at least 30 embryos, otherwise only 10 embryos were taken from the pouch) of each male. We dried the embryos for 36 hours at 70°C, weighed them on a Sartorius LE26P microbalance to the nearest 0.01 mg and calculated the average embryo weight.

By measuring the absolute embryo mortality we can measure how many embryos that might nutritionally be available for the brooding male (each absorbed embryo is one package of nutrients). However, the relative embryo mortality is relevant to use when comparing embryo mortality between partial and full broods: For example, 50 dead embryos might mean a relative mortality of 1 (all embryos dead) for a PF male, but only 0.5 (half of the embryos dead) for a FF male.

We used the hepatosomatic index (HSI) to estimate final male condition at the end of the experiment. The fish liver is often highly responsive to feeding conditions [48–50], and HSI indicates the energetic status of the fish [51–52]. It is calculated as (liver dry weight x 100) x body dry weight^-1^. At dissection, the liver was separated from the rest of the body. Both liver and body (without the brood) were dried and weighed in the same way as described above for embryos.

The statistical analyses were done in SPSS 21 and 22 (SPSS, Inc., Chicago, IL, USA). Parametric tests were used whenever the data met the assumptions of normality and homogeneity of variances. All mean values are reported with ± standard error (SE). In analyses of covariance (ANCOVA) non-significant interaction terms were removed, starting from the highest order.

## Results

### Brood size, embryo mortality and reproductive outcome

The number of males that showed any embryo mortality did not differ significantly between the partially filled (PF) and fully filled (FF) males (χ^2^ = 0.54, N = 74, P = 0.46). However, there was a significant effect of brood size treatment on both absolute embryo mortality (ANCOVA: treatment: F_1,71_ = 11.81, P < 0.001, days brooding: F_1,71_ = 1.67, P = 0.20, interaction NS) and relative embryo mortality (ANCOVA: treatment: F_1,72_ = 6.75, P = 0.010, days brooding: F_1,71_ = 1.90, P = 0.17, interaction NS; Fig 2). Although number of days brooding varied in both groups, it did not affect the outcome of the analysis.

Among the males that survived to the end of the experimental period, the absolute embryo mortality was on average 2.5 embryos (relative embryo mortality 6%) in the PF male group and 10.5 embryos (relative embryo mortality 12%) in the FF male group. Yet, neither of these differed statistically due to high variances, and again, number of days brooding did not affect the outcome (absolute embryo mortality: ANCOVA: treatment: F_1,33_ = 3.58, P = 0.067, days brooding: F_1,33_ = 0.53, P = 0.47, interaction NS; relative embryo mortality: ANCOVA: treatment: F_1,33_ = 1.35, P = 0.25, days brooding: F_1,33_ = 0.18, P = 0.68, interaction NS).

At the end of the experiment, males in the FF group still brooded significantly higher numbers of embryos compared to males in the PF group (all males: one-way ANOVA: treatment: F_1,72_ = 16.04, P < 0.001; survivors only: one-way ANOVA: treatment: F_1,34_ = 17.58, P < 0.001), despite the higher absolute and relative embryo mortality in FF broods (Fig 2). Yet, if we compare the average outcome for a PF *vs*. a FF-male, by combining the mean number of surviving embryos with the probability that the brooding male will survive (see below), the reproductive outcome is similar for the two groups (probable reproductive outcome: PF: 45 embryos/male x 0.63 male survival probability = 28 embryos/male; FF: 77 embryos/male x 0.39 male survival probability = 30 embryos/male).

### Male initial condition and embryo mortality

The males that died prematurely showed a significantly higher absolute and relative embryo mortality than males that survived until the end of the experiment (absolute embryo mortality: one-way ANOVA: male survival: F_1,73_ = 12.40, P = 0.001; relative embryo mortality: one-way ANOVA: male survival: F_1,73_ = 13.57, P < 0.001). Focusing on the group of males that survived and using treatment as factor, and initial male condition (width by length residuals) and male length as covariates, we found less embryo mortality in males that were in better initial condition, but embryo mortality was unaffected by male length. This was the case both for absolute and relative embryo mortality (absolute embryo mortality: ANCOVA: treatment: F_1,32_ = 3.86, P = 0.058, initial male condition: F_1,32_ = 7.92, P = 0.008, male length: F_1,32_ = 0.34, P = 0.57, interactions NS; relative embryo mortality: ANCOVA: treatment: F_1,32_ = 1.13, P = 0.30, initial male condition: F_1,32_ = 5.86, P = 0.021, male length: F_1,32_ = 0.12, P = 0.74, interactions NS).

### Embryo weight

Among the males that survived to the end of the experiment, embryo weight was negatively affected by initial male condition and absolute embryo mortality, positively affected by how long time the embryos had been brooded, but unaffected by brood size treatment (ANCOVA: treatment: F_1,31_ = 0.12, P = 0.73, initial male condition: F_1,31_ = 4.45, P = 0.043, absolute embryo mortality: F_1,31_ = 4.84, P = 0.035, days brooding: F_1,31_ = 5.23, P = 0.029, interactions NS). Hence, embryo weight was higher in broods with low embryo mortality and long brooding time, but males in high initial condition brooded embryos of lower weight in the end.

### Brood size, male final condition (HSI) and male survival

Fewer PF males than FF males died prematurely (14 of 36 = 39 percent vs. 24 of 38 = 63 percent, χ^2^ = 4.36, N = 74, P = 0.037). However, the survival curve was similar for both treatments (Kaplan Meyer: χ^2^ = 2.23, N = 74, P = 0.14). A logistic regression model on whether a male survived or not as dependent, with treatment as factor and male length, initial male condition, final male condition (HSI) and absolute embryo mortality as covariates, was significant (Nagelkerke r^2^ = 0.66, χ^2^ = 50.37, df = 5, P < 0.001). Male survival covaried positively with HSI (Wald = 13.92, df = 1, P < 0.001), whereas the other variables were not significant (treatment: Wald = 2.57, df = 1, P = 0.11, male length: Wald = 1.52, df = 1, P = 0.22, initial male condition: Wald = 1.80, P = 0.18, absolute embryo mortality: Wald = 2.55, df = 1, P = 0.11). Consistent with this, HSI was significantly lower among the males that died, but did not differ between PF and FF males (two-way ANOVA: treatment: F_1,70_ = 0.002, P = 0.97, male survival: F_1,70_ = 43.67, P < 0.001, interaction: NS).

## Discussion

In natural populations of broad-nosed pipefish, females produce more eggs than males can care for. Females therefore compete among themselves for males to mate with and males are in general mated to their full pouch capacity [30, 32–33]. In the study presented here, males that were allowed to fill up their brood pouches fully had greater numbers of developing offspring at the end of the experiment, than did males that were only allowed to fill their brood pouches partially, showing there are important benefits to filling up the brood pouch. Still, we also found important negative effects of a large brood size on both embryo survival and male survival, but not on embryo weight. In addition, embryo survival was lower when the brooding males were in poor initial condition. Males that died were in lower final condition, and these males also showed higher embryo mortality. Thus, as discussed in more detail below, these results indicate multiple costs of paternal care related to brood size, often mediated via male condition.

Absolute embryo mortality was higher in full broods than in partial broods (Fig 2), although among the males that survived to the end of the experiment (who also were in better condition) this difference was not significant. Similarly, the relative embryo mortality in full broods was on average twice as high as in partial broods, and this was significant for all experimental males, but not when analysing survivors separately. Thus, the strongest effect of brood size on embryo mortality was found among the males that themselves did not survive to the end of the experiment. Although this effect may have been accentuated by the experimental situation, compared to the field, it nevertheless suggests that for males in poor health, brood size matters. In nature, such effects are likely to be more important during bad years and in marginal or anthropogenically degraded habitats, than for healthy fish in pristine waters. As reviewed by Mock [9], offspring survival has been found to be negatively affected by initial brood size in many taxa. The results of our study indicate that number of offspring may influence embryo mortality also in pipefish, at least for males in poor health. It also indicates that care provided through the brood pouch is not a shareable resource. In particular, given that males of the broad-nosed pipefish, during their pregnancy, provide both nutrients and oxygen to the developing offspring [26, 28], it is reasonable to assume that the result of higher absolute and relative survival for embryos in small broods is due to fewer embryos sharing these resources. Similarly, in barn swallows (*Hirundo rustica*), a reduction in clutch size before hatching resulted in higher hatching success and shorter incubation time among the remaining offspring [53]. In other cases, the effect of a reduced brood size on the remaining offspring’s fitness may be weaker or absent due to a corresponding decrease in parental care, as found in the smallmouth bass [54].

We found that a large brood size reduces the survival of caring males. Broad-nosed pipefish can live for two or possibly three years, and breed multiple times during each reproductive season [24, 32–33]. Therefore, reduced survival represents a substantial fitness cost beyond the current brood. The fish in the two treatments initially had similar length, width and initial condition and were treated the same way. It is therefore unlikely that the effect on male survival would be caused by any other factor than the manipulated brood size. Altered parental survival due to brood size manipulation has also been found in the kestrel (*Falco tinnunculus*) [55] and a dung beetle (*Onthophagus taurus*) [56]. Furthermore, increased brood size may lead to higher level of parasite infection and immunoresistance, as shown in the great tit (*Parus major*) [57]. Interestingly, in the broad-nosed pipefish males are known to have a more active immune response than females [58], and it is possible that resource allocation towards such an upregulated immune response benefit paternal survival, at a cost of embryo survival.

At the end of the experiment, there were significantly larger numbers of developing offspring in full than in partial broods, despite higher embryo mortality in full broods. However, when taking the likelihood of paternal survival into account the outcome of the two treatment groups is relatively similar, with a probable reproductive output of 28 and 30 embryos/brood for the partially and fully filled treatments, respectively. Males in this population typically fill their brood pouches completely. As long as the male survives, this should be adaptive, given the higher number of developing embryos. Possibly, by filling their brood pouch fully, males take the chance to produce many young if conditions are beneficial [59]. That available resources matter has been demonstrated in the Gulf pipefish (*Syngnathus scovelli)* where pregnant males in a high food treatment allocated resources both to brooding and growth, whereas those in a low food treatment sacrificed somatic growth and invested into the current brood [60]. Another option that a paternal pipefish can benefit from is the ability to absorb nutrients that originate from former embryos in the pouch [40]. In our experiment, small broods were created experimentally. However, under more natural circumstances, the option of nutrient uptake suggests an obvious benefit of starting with a large brood, which size may then be reduced over the brooding period. Indeed, in *S*. *typhle* it is common for males to brood about 20–30% fewer embryos in the end than initially supported [35–38, 61].

When there are spatial limitations for a brood, embryos in larger broods are typically brooded at a higher density. In the broad-nosed pipefish, however, half a brood does not result in twice the space per embryo, since males fill their pouches from the bottom of the pouch and seal off any empty space in the upper parts of the pouch. Therefore, embryo density varied less than embryo number, although we cannot completely exclude that it contributed to the higher relative survival of the young brooded in half-filled pouches. Higher embryo survival at lower densities has been found in other pouch and nest brooding fishes [62–63] and has usually been assigned to oxygen availability[11, 64]; but see [62–63, 65].

Males in better initial condition showed lower embryo mortality. The different aspects of care in pipefish, including osmoregulation and provisioning of oxygen and nutrients, can be expected to be energetically costly to the brooding males [24, 66, 67]. Hence, it is likely that males in lower initial condition have fewer resources available to start with and are less able to provide good care, therefore leading to higher embryo mortality. This is consistent with parental care in general being an energetically demanding task, causing caring parents to be particularly constrained energetically during the breeding period [22]. Parental care has also been found to decrease HSI in the paternal caring stream goby (*Rhinogobius sp*.) [68] and in the rock bass (*Ambloplites rupestris*) increased brood size resulted in decreased body mass [15]. In our experiment, however, male final condition (HSI) did not differ between brood size treatments. While HSI was positively correlated to male survival, inital condition was unrelated. Similarly, higher mortality among caring males due deteriorating condition has been found in other fishes, such as *Cottus gobio* and *Gasterosteus aculeatus* [23, 69–70].

Consistent with previous findings for this population [44], males with partially or fully filled brood pouches produced embryos of similar weight. This result indicates that it might be more important for a resource limited male to maintain embryo weight than embryo number, similarly to what has been suggested in earlier studies of this fish [35, 71]. Such a priority of weight over number is likely to be adaptive, because the survival of newborn pipefish is positively related to their weight when facing predation by anemones [61]. Nevertheless, our results show that males in good initial condition brooded embryos of a lower weight and had a lower embryo mortality, and as a consequence would brood more embryos. Males in better initial condition might be more optimistic about the future (*sensu* [59]), which might lead to a trade-off between embryo number and weight, a trade-off that becomes evident only in relation to initial condition and not when the treatments are compared.

In conclusion, comparing our brood size treatments, carried out in a semi-natural setting including pathogens, we found that benefits of having a large brood to start with were counterbalanced by costs in terms of higher embryo mortality and paternal mortality. Importantly, these results contribute to a better understanding of brood reduction, which is reported in several species of pipefish. Furthermore, we found that embryo mortality covaried with initial condition of the brooding male while male survival covaried with final condition. Thus, our results suggest that paternal care in the form of a male pregnancy comes with multiple costs and trade-offs that are related to brood size and resource availability.

## Supporting Information

S1 File Original data of the study. All lengths and widths are in mm, all weights are in g.
(XLSX)
